# Is Motor Function in MCI Related to Caregiver Ratings of Cognition and Everyday Function?

**DOI:** 10.1093/geroni/igaf122.3799

**Published:** 2025-12-31

**Authors:** Kyoung Shin Park, Jeongwoon Kim, Kayci Vickers, Emily Giannotto, Jacquelyn Thelin, Liselotte De Wit, Amy Rodriguez

**Affiliations:** Emory University School of Medicine, Atlanta, Georgia, United States; Emory University School of Medicine, Atlanta, Georgia, United States; Emory University School of Medicine, Atlanta, Georgia, United States; Emory University School of Medicine, Atlanta, Georgia, United States; Emory University School of Medicine, Atlanta, Georgia, United States; Emory University School of Medicine, Atlanta, Georgia, United States; Emory University School of Medicine, Atlanta, Georgia, United States

## Abstract

Mild cognitive impairment (MCI) often affects daily functioning, which is typically assessed by subjective caregiver ratings. Previous research shows that caregiver ratings may be biased by individual factors, such as caregiver burden and stress. We aimed to extend this work by further examining whether decline in motor function in MCI also influences caregiver perceptions, potentially leading to lower ratings of patients’ cognition and everyday function. A cross-sectional study was conducted with 124 MCI patient-caregiver dyads (patients: age=74.7±6.6 years, 49% female; caregivers: age=65.6±13.3 years, 76% female). Caregivers rated MCI patients’ cognition and daily function using the Everyday Cognition Scale (ECog-39) and Functional Activities Questionnaire (FAQ) and completed the Zarit Burden Interview Short Form (ZBI-12) and NIH Toolbox Perceived Stress Scale (PSS). Patients completed the Montreal Cognitive Assessment (MoCA) and motor assessments (10-meter walk, handgrip strength, and 30-second chair stands). Multiple linear regressions were fitted while controlling for patient (age, sex, education, MoCA) and caregiver (age, sex) covariates. Consistent with the prior work, higher caregiver burden and stress were associated with lower caregiver ratings on ECog-39 (ZBI-12: B = 0.86, p<.001; PSS: B = 0.94, p<.001) and FAQ ratings (ZBI-12: B = 0.03, p<.001; PSS: B = 0.22, p=.026). In contrast, walking speed, grip strength, and chair stands were not associated with ECog-39 and FAQ ratings (all p>.10). Caregivers’ subjective ratings of MCI patients’ cognition and daily functioning were more closely tied to their own burden and stress than to patients’ motor performance. These findings highlight the need to consider caregiver factors when interpreting subjective functional ratings in MCI.

